# Drivers of dune formation control ecosystem function and response to disturbance in a barrier island system

**DOI:** 10.1038/s41598-024-61741-9

**Published:** 2024-05-18

**Authors:** Alexander B. Sabo, Michael R. Cornish, Max C. N. Castorani, Julie C. Zinnert

**Affiliations:** 1 Department of Biology, Virginia Commonwealth University, Richmond, VA USA; 2 Department of Environmental Sciences, University of Virginia, Charlottesville, VA USA

**Keywords:** Ecology, Environmental sciences

## Abstract

Barrier islands are landscape features that protect coastlines by reducing wave energy and erosion. Quantifying vegetation-topographic interactions between adjacent habitats are essential for predicting long-term island response and resilience to sea-level rise and disturbance. To understand the effects of dune dynamics on adjacent interior island ecosystem processes, we quantified how sediment availability and previous disturbance regime interact with vegetation to influence dune building and ease of seawater and sediment movement into the island interior on two US mid-Atlantic coast barrier islands. We conducted field surveys of sediment accretion, vegetative cover, and soil characteristics in dune and swale habitats. Digital elevation models provided assessment of water flow resistance from the mean high water mark into the island interior. We found that geographic location impacted sediment accretion rates and *Panicum amarum* (a species increasing in abundance over time in the Virginia barrier islands) accreted sediment at a significantly lower rate compared to other dune grasses. Dune elevation impacted the ease of seawater flow into the island interior, altering soil chlorides, annual net primary productivity, and soil carbon and nitrogen. Our work demonstrates the importance of incorporating biological processes and cross-island connectivity into future scenario modeling and predictions of rising sea-levels and increased disturbance.

## Introduction

Barrier islands protect 10% of global shorelines and 30% of United States Atlantic and Gulf coasts^1,2^. These landforms provide a variety of services to both humans and surrounding ecosystems including reduction of wave energy and storm surge, carbon sequestration, provisioning habitat for a multitude of organisms, as well as recreation and tourism^3–5^. Because barrier islands have low topography and are composed of unconsolidated substrates, they are susceptible to disturbance driven by storm and tidal induced overwash^6^. Increasing rates of sea-level rise impact barrier islands, resulting in land area loss, island migration, and changes to overall island habitats, altering the associated ecosystem services.

Barrier islands are highly dynamic and undergo changes on many spatiotemporal scales. On the largest of these scales (barrier islands and island chains), sediment movement and ocean currents can drive broad change including island migration or rotation^7–9^. This sediment movement can impact a barrier island system regionally and can be influenced by events many kilometers away (e.g. inlet formation/dredging, groin or jetty construction, beach nourishment)^9–11^. Large-scale change on barrier islands is driven by daily wave action, tides, and episodic storm events. The daily press of wave action can lead to gradual erosion and landscape change, while the rapid pulse of a storm event can cause sudden shifts in island ecology and geomorphology^12–14^. Along the US Atlantic coast, these storms come in the form of hurricanes and nor’easters. Storm events drive the movement of sediments, changing island shape and causing overwash and island migration^3,15,16^. With changing climate, storms will increase in severity and frequency^17^ resulting in further changes to barrier island systems.

On smaller scales of individual islands and habitats, plant species composition and topography impact how the island responds to storm and overwash events^6,8,14^, affecting island resistance and resilience. Here, barrier island resilience is defined as the ability of the island to maintain elevation relative to sea-level rise, which can result in island migration and shifting island habitats^8,18^. Barrier island resistance occurs when an island resists changes that are driven by sea-level rise and severe weather, but over time may result in higher rates of shoreface erosion and loss of sediment to build up island interior or backbarrier marsh elevation^8^. Climate change may alter vegetation and sediment dynamics, such as species range shifts due to warming temperatures^19,20^ and island erosion/migration due to sea-level rise. However, future scenarios are highly uncertain^21^ and quantifying vegetation-sediment interactions across the landscape could improve modeling efforts.

### Dune building

Dune plants, often grasses, modify the physical environment by trapping moving sediment. Plant growth and sand accretion result in dune formation, land stabilization, and a reduction of wave energy/erosion^22–25^. In coastal dunes, grasses function as ecosystem engineers, modifying and enhancing the topography of the barrier island landscape^14,24^. Recent research has focused on the topographic and vegetation interactions that occur across barrier island habitats^26,27^. Dune elevation modifies surrounding island ecosystems, impacting adjacent swale habitat (e.g., interior low lying elevation) succession and state transitions between upland and marsh habitat^8,28^.

Dune building is impacted by disturbance in the form of severe weather and overwash events that can result in burial of dune plants or large-scale erosion^3^. Disturbance events can also result in the temporary reset of a dune community^29^. In places where disturbance is more frequent, the dune community may not have sufficient time to recover between events, preventing new dune formation^29,30^. Although recent studies have documented aspects of how species interact with sediment^31,32^, studies examining natural dune grass populations and sediment capture over time are lacking. Quantifying interactions between dune building grasses and sediment movement at different locations across barrier islands will enhance predictions of future conditions created by storms and sea-level rise disturbance^6,14^.

### Barrier island habitats

**Figure 1:**
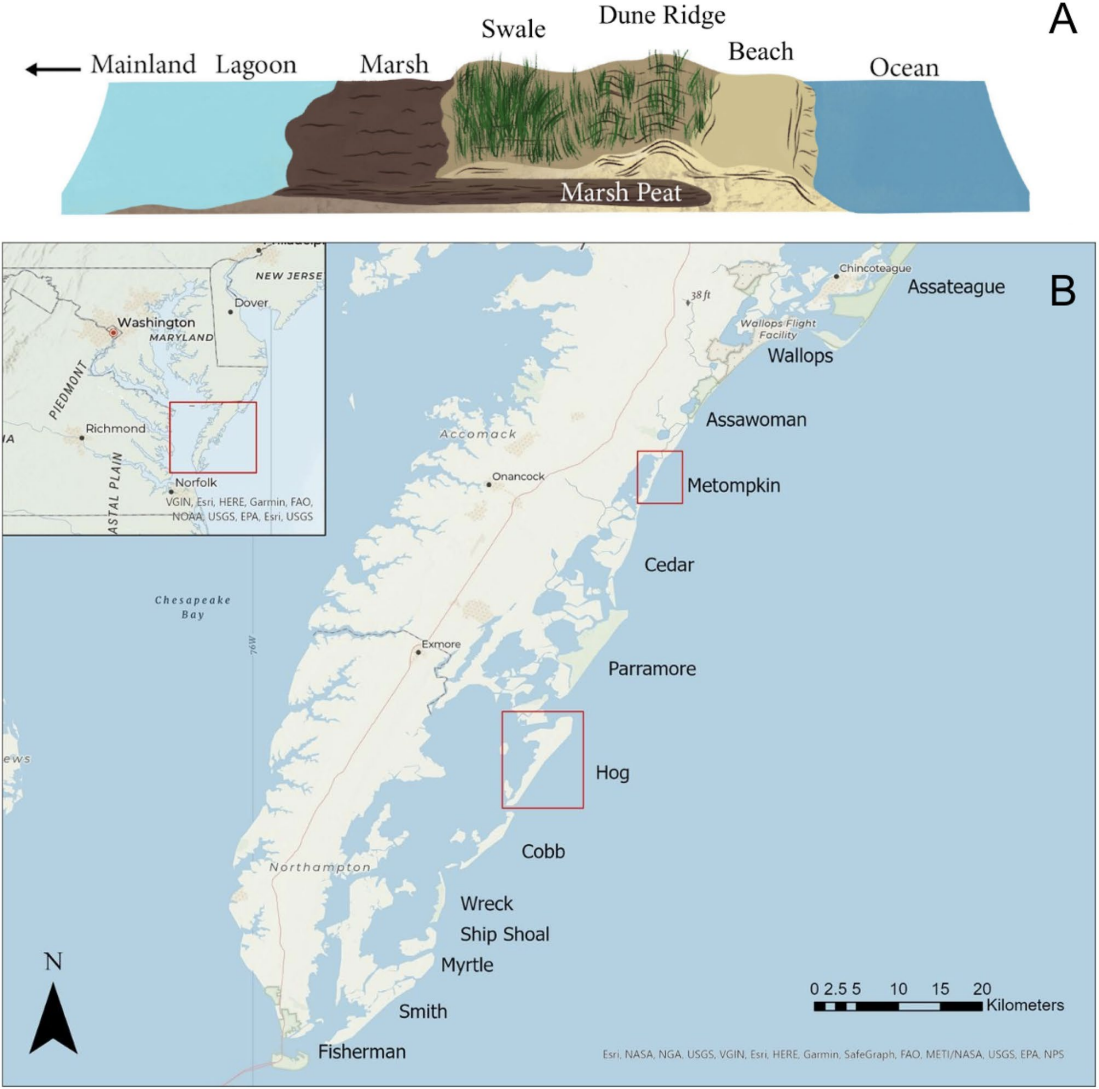
(**A**) Cross-section of a typical barrier island. Image created by Julia Yee. (**B**) The barrier islands of the Virginia Coast Reserve. Islands studied marked in red. Study areas are located on the southern ends of both Hog and Metompkin Islands.

Barrier islands are composed of distinct habitats including beach, dune, swale, and back-barrier marsh (Fig. 1A). Variability in dune shape and size created by different dune building grasses can impact interior island processes across the barrier island ecosystem. These differences lead to distinct vegetative zones on barrier islands, primarily consisting of dune and swale communities^29^. As new dune formation occurs, embryonic dunes (i.e. hummocks) will coalesce into foredunes, later forming dune ridges and additional swale habitats^33^. The formation of these separate habitats results in differing elevations and distance from shoreline, with plant species uniquely adapted to living in specific conditions^34^. These topographic separations affect species colonization which can lead to further habitat modification.

Islands dominated by lower, hummock dunes are often impacted more frequently by disturbance events as dune ridges do not form^16,23^. This leads to swale habitat that is similar to the surrounding dune and beach^14,29^. Conversely, islands dominated by taller, linear dune ridges are more protected from disturbance events and the swale habitat is less frequently impacted leading to a swale habitat that is markedly different from dune and beach habitats^29^. These differences in community structure have the potential to impact nitrogen (N) and soil carbon (C) storage across the barrier island landscape^35^. Thus, differences in dune topography and the vegetation of adjacent swales have large-scale impacts on multiple components of barrier island ecosystem function^8,36^.

The study area for this research was two islands on the Virginia Eastern Shore, located within the Virginia Coast Reserve (VCR). The VCR is a collection of islands spanning from Assateague Island in the north to Fisherman Island in the south^12^ (Fig. 1B). This region was designated by the National Science Foundation as a Long-Term Ecological Research site and is managed by The Nature Conservancy^12^. Since the evacuation of Broadwater, Hog Island in the 1930s, these islands have been primarily uninhabited. This has created a vast undeveloped barrier island system with limited direct human influence^12^. Dominant dune building grasses in the region that may differ in sediment accretion include *Ammophila breviligulata*, *Spartina patens*, and *Panicum amarum* (hereafter referred to by genus)^14,29,31^.

In order to understand the effects of dune dynamics on adjacent interior island ecosystem processes (i.e., carbon, vegetation annual net primary productivity [ANPP]), we quantified: (1) how sediment availability and disturbance interact with dominant dune grasses to influence rates of sediment accretion and soil characteristics over time and (2) how dune topography-disturbance interactions influence swale vegetation and soil characteristics during 2020–2022. We focused on Hog and Metompkin Islands, two US mid-Atlantic coast barrier islands in Virginia that differ in disturbance intensity based on prior landscape change^8,29^ (Fig. 1B). We hypothesized that increased rates of dune building would provide protection for the adjacent swale habitat from seawater, increasing vegetative productivity and the potential for carbon accumulation. In fall 2020, we established cross-shore transects to quantify natural dune vegetation, and starting in fall 2021 additional plots in dune and swale where vegetation cover and sediment accretion were monitored over time. Soils were sampled for organic matter content, carbon, nitrogen, soil chlorides, and bulk density. Digital elevation models were collected in 2020 and 2022 to quantify ease of sediment and water movement into the island interior. This was done utilizing least cost path analysis and storm surge analysis, providing quantitative measurements of water movement in ArcGISPro.

## Results

Ecosystem interactions, dune development, and productivity varied between our study sites, Hog and Metompkin Islands. These differences emerge as a result of many complex and interacting factors including sediment availability, plant species composition, and dune development. As dunes accrete sediment more quickly on Hog, the interior swale habitat is better protected resulting in higher ANPP, soil C and N, and lower soil salinity. These effects are reflected in our ground-truthed data, as well as GIS analysis.

### Dune sediment accretion

**Figure 2:**
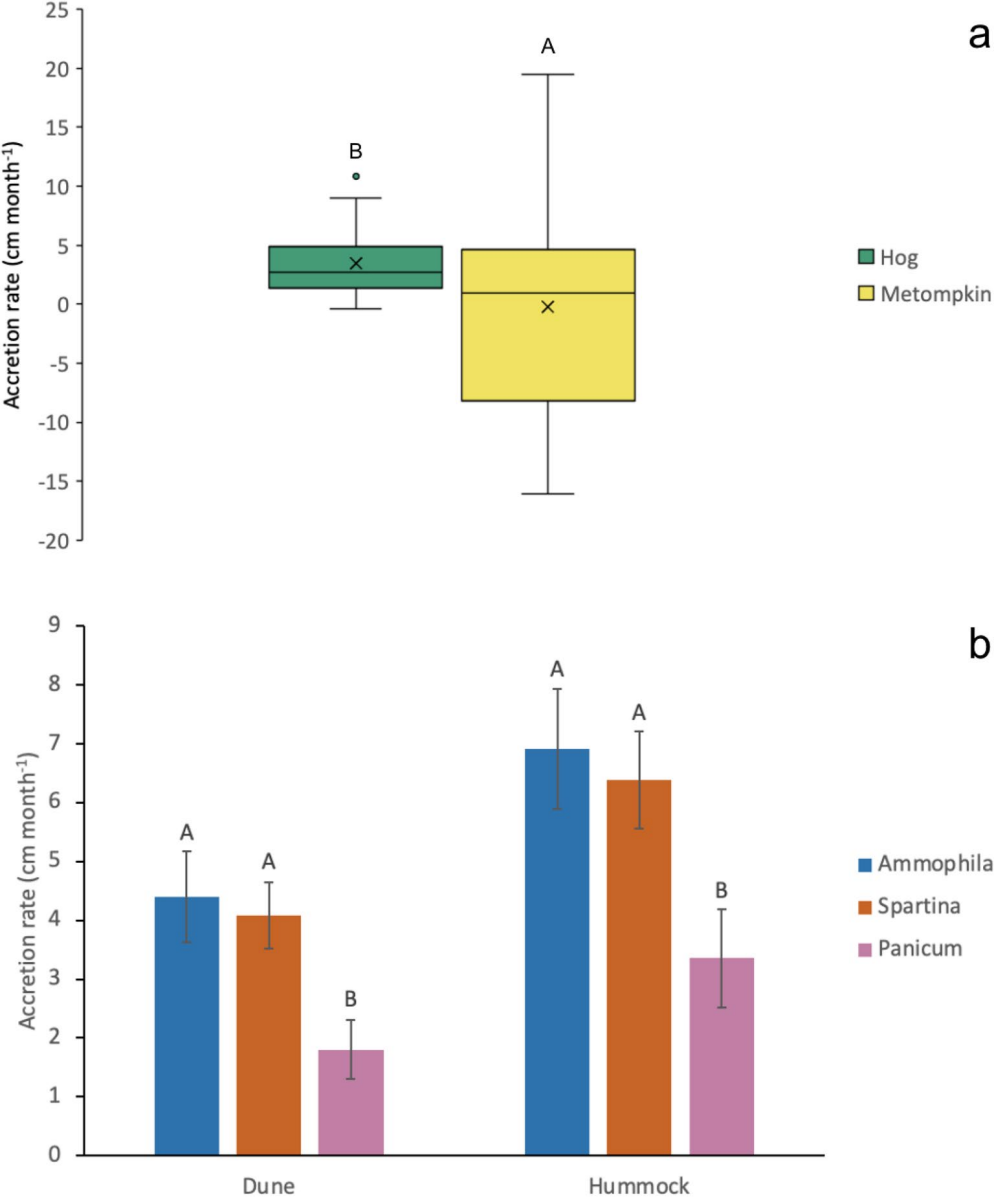
(**a**) Mean sediment accretion rate ± standard error (cm month^−1^) for dunes on Hog and Metompkin islands. Letters indicate Metompkin accretes significantly less sediment than Hog. (**b**) Mean sediment accretion rate ± standard error (cm month^−1^) by habitat and dominant species on Hog. Letters indicate that *Ammophila* and *Spartina* are not significantly different in the dune or hummock, but *Panicum* accretes significantly less sediment.

Dune sediment accretion rates were significantly different between the two barrier island study sites. Accretion was higher on Hog (3.4 ± 0.4 cm month^−1^) compared to Metompkin (− 0.2 ± 1.3 cm month^−1^; F = 5.88; p = 0.017; Fig. 2A, Table S1). 9 plots lost elevation on Metompkin and were below the high tide line by November 2022, resulting in high accretion variability. Additional dune plots on Metompkin remained above high tide but transitioned to open beach. Because of the wide variation in sediment accretion and erosion on Metompkin, no species effects on accretion were seen.

On Hog, monthly sediment accretion rates in newly formed dune hummocks were 35% higher than in plots on the existing dune face (F = 12.70; p = 0.0007; Fig. 2B, Table S2). Within these two habitats, three dominant dune building grasses were identified and found to accrete sediments at different rates; *Ammophila* and *Spartina* were two times higher (~ 5.0 cm month^−1^) compared to *Panicum* (F = 12.09; p < 0.0001; Fig. 2B). Sediment accretion rates were highest in fall (6.8 ± 0.7 cm month^−1^) and lowest in winter (3.1 ± 0.3 cm month^−1^; F = 23.44; p < 0.0001). No species-specific effects were observed on Metompkin.

### Dune vegetation and soil characteristics

*Ammophila*, *Spartina*, and *Panicum* were dominant dune grass species on both islands, with relative cover of *Panicum* increasing from 2020 to 2022 (Fig. S1). Plant cover on dunes was significantly higher (> 40%) on Hog compared to Metompkin (F = 43.54; p < 0.0001; Table 1, Table S3). In dune plots, plant cover was significantly lower during fall 2022 on Metompkin, coinciding with foredune plots transitioning to open beach (F = 11.20; p < 0.0001; Fig. S2). Stem numbers in dune plots were twice as high on Hog as compared to Metompkin (Table 1; F = 12.54; p = 0.0008; Table S4). Species-specific differences were observed in stem number, with *Spartina* having the most stems (42 ± 8) compared to *Ammophila* and *Panicum* (18 ± 3 and 14 ± 2; F = 5.94; p = 0.0045). ANPP did not differ between Hog and Metompkin dunes (Table S5).

**Table 1:** Vegetation and soil characteristics of Hog and Metompkin islands by habitat (mean ± standard error).

Metric	Hog	Metompkin
Dune		
Percent cover	**16 ± 1**	**9 ± 1**
Stem count (0.25 m^2^)	**35 ± 5**	**14 ± 3**
Soil chlorides (ug g^−1^)	**44 ± 14**	**180 ± 32**
Organic matter (%)	0.3 ± 0.02	0.3 ± 0.03
Soil carbon (g m^−2^)	**66 ± 3**	**38 ± 7**
Soil nitrogen (g m^−2^)	**6 ± 3**	**2 ± 0.2**
Bulk density (g cm^−3^)	**1.3 ± 0.01**	**1.4 ± 0.01**
ANPP (g m^−2^ year^−1^)	261 ± 39	235 ± 67
Swale		
Percent cover	**52 ± 5**	**13 ± 2**
Soil chlorides (ug g^−1^)	**14 ± 5**	**90 ± 31**
Organic matter (%)	**0.42 ± 0.03**	**0.26 ± 0.04**
Soil carbon (g m^−2^)	**82.9 ± 10.9**	**41.3 ± 4.6**
Soil nitrogen (g m^−2^)	**9.7 ± 1.1**	**4.6 ± 0.3**
Bulk density (g cm^−3^)	**1.3 ± 0.02**	**1.4 ± 0.02**
ANPP (g m^−2^ year^−1^)	**294 ± 45**	**191 ± 23**

Significant differences between islands are indicated in bold.

In the dune, n = 15 per island, per season. In the swale, n = 5 per island, per season.

**Figure 3:**
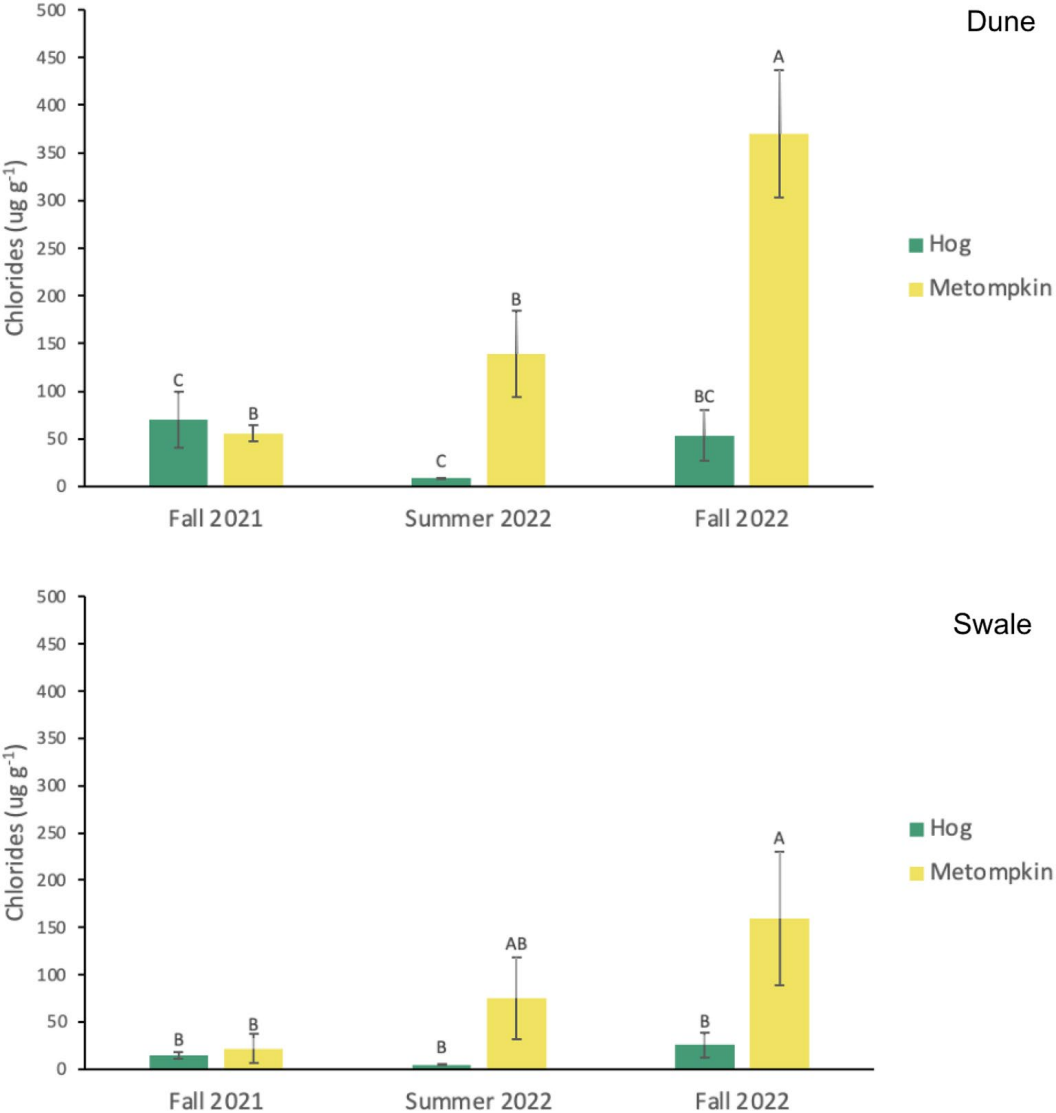
Mean seasonal chlorides ± standard error (ug g^−1^) for dune and swale habitats on Hog and Metompkin. Within each graph, letters indicate significant statistical differences in soil chlorides across islands and time. Shared letters denote no mean difference in groups. Overall, Hog has lower chlorides than Metompkin in both habitats, but it is seasonally variable.

Dune soil characteristics varied spatially and temporally. Organic matter content was lowest in summer 2022 (0.20 ± 0.05%) and highest in fall 2021 on Hog (0.34 ± 0.02%; F = 4.44; p = 0.015; Table S6). Soil chlorides were 55% higher on Metompkin compared to Hog (F = 61.03; p < 0.0001; Table 1; Fig. 3; Table S7) and highest in fall 2022 (F = 15.66; p < 0.0001). Soils were less compact on Hog dunes (F = 19.02; p < 0.0001; Table S8) with significantly higher C and N (42% and 66% respectively) relative to Metompkin dune soils (Table 1).

### Cross-island connectivity

**Figure 4:**
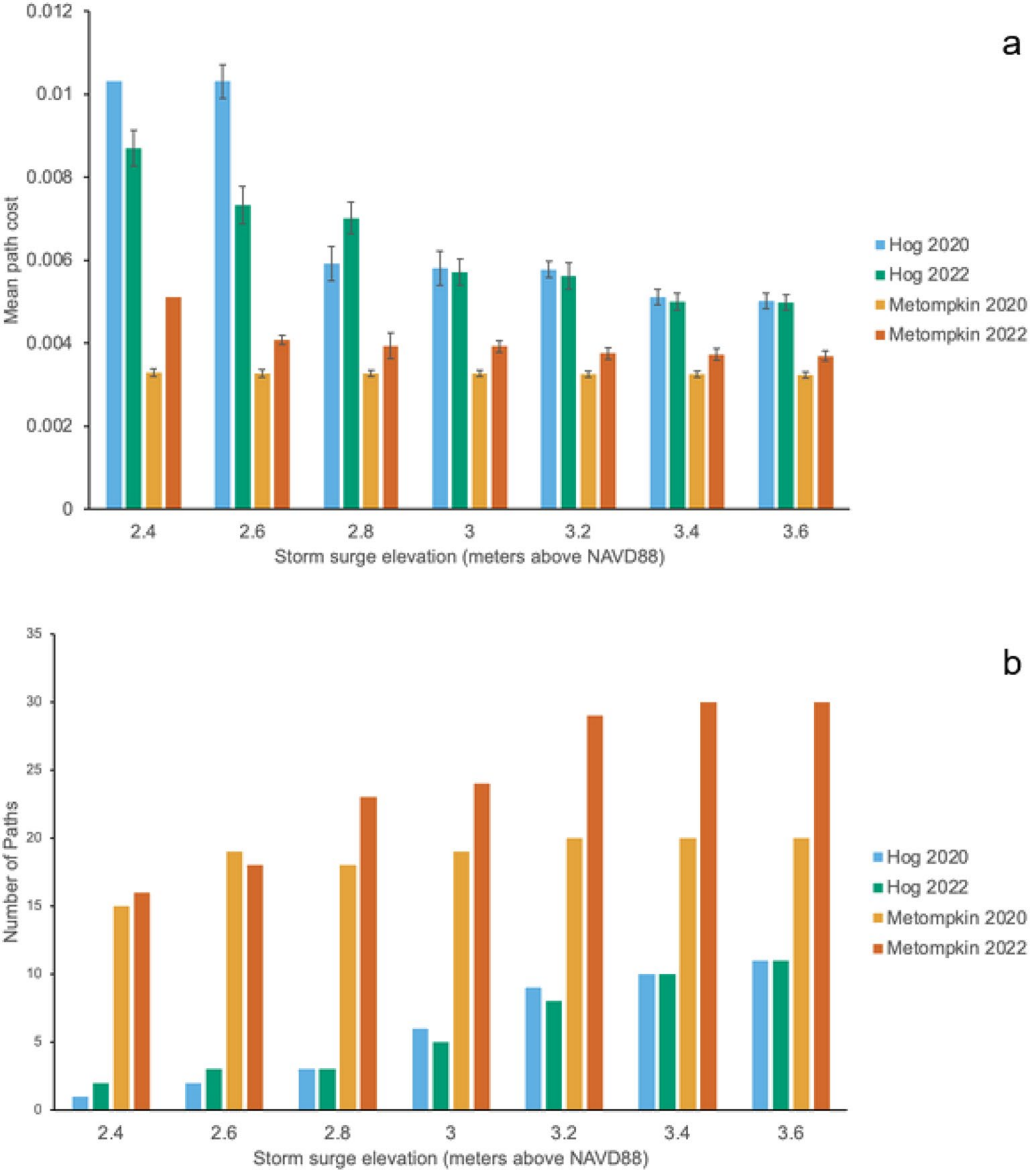
(**a**) Mean cost path of storm surge for both islands in 2020 and 2022 from least cost path analysis. Higher values indicate more resistance to seawater flow across the dune system. (**b**) Number of paths across the dune ridge at different storm surge elevations for both islands in 2020 and 2022.

Elevation differed between islands, habitats, and over time. Hog dunes exhibited the highest overall elevation (2.33 ± 0.11 m) followed by Hog swale (1.99 ± 0.09 m; F = 4.32; p = 0.039; Table S9). There was no difference between Metompkin dunes (1.75 ± 0.09 m) and swale (1.74 ± 0.05 m). In 2022, Metompkin had the lowest overall elevation (1.51 ± 0.10 m), while Hog had the highest (2.31 ± 0.13 m; F = 6.99; p = 0.001; Table 2). Average path cost (resistance against movement across the landscape) was 7% higher on Hog compared to Metompkin (F = 855.09; p < 0.0001; Fig. S3; Table S10), creating more topographical resistance of seawater and sediment traveling from the beach to the interior swale. Storm surge analysis showed that dunes on Hog are harder to breach and provide better protection for the swale due to being taller and more continuous. A storm surge of 2.4 m showed higher overwash occurrence (determined by number of paths breaching the dune) on Metompkin than Hog (Fig. 4). There were less than 5 breached dune points on Hog at storm surge < 3.0 m. As storm surge heights increased, Metompkin had significantly more dune breach points (F = 8.14, p < 0.0001; Table S11).

**Table 2:** Mean ± standard error elevations (m) for dune and swale habitats on Hog and Metompkin.

	2020	2021	2022
Hog			
Dune	2.42 ± 0.21	2.10 ± 0.16	2.47 ± 0.18
Swale	2.11 ± 0.16	1.71 ± 0.12	2.15 ± 0.18
Metompkin			
Dune	1.82 ± 0.09	2.07 ± 0.11	1.36 ± 0.22
Swale	1.77 ± 0.09	1.71 ± 0.07	1.76 ± 0.09

### Swale vegetation and soil characteristics

Grassland swale plant cover was 120% higher on Hog compared to Metompkin (Table 1; F = 45.21; p < 0.001; Table S12). Cover on both islands was highest at the end of the growing season (August; F = 2.95; p = 0.04). Swale ANPP was 44% higher on Hog (F = 11.21, p = 0.001; Table 1; Table S13). As in the dunes, soil characteristics varied spatially and temporally. Swale soil chlorides were 36 times higher on Metompkin and highest in summer and fall 2022 (F = 4.41; p = 0.0188; Table 1; Fig. 3; Table S15). Metompkin swale soils had higher bulk density (F = 13.38; p = 0.0008; Table S16) with lower organic matter content than Hog (F = 6.38; p = 0.0185; Table 1; Table S14). Soil C and N stocks in the swale were 51% and 48% higher on Hog (Table 1).

## Discussion

Although we understand the ecological processes that lead to dune formation and succession on barrier islands, reducing knowledge gaps about how adjacent island ecosystems interact and how dune size and continuity impact interior island function can enhance future predictions of island dynamics^5,30,37^ due to expected increases in sea-level rise and disturbance events (i.e., hurricanes, nor’easters)^17,18^. Here, we demonstrated how dominant dune grass species accrete sediment at different rates depending on landscape location and how dune dynamics differ on two Virginia barrier islands that result in different interior swale ecosystem function (ANPP, soil C and N) based on ease of sediment and water movement.

Increased protection offered by a prominent, linear dune ridge on Hog allowed for higher swale productivity, increased soil C and N, and decreased soil chlorides. This was reflected in plant and sediment samples, as well as least cost path and storm surge analyses. Connectivity of adjacent habitats should be incorporated into modeling barrier island evolution and C variability under different climate change scenarios. For example, recent modeling has incorporated shrub expansion into barrier island evolution^36^ based on known dune elevations that determine shrub presence^28^. Continued changes in climate warming, sea-level rise, and sediment dynamics impact species distributions that lead to differences in dune building and overall community composition^20,29,38^. Quantifying sediment movement across the dune and how this affects the landward vegetation can reduce uncertainties associated with future scenario modeling that rely on predicting habitat change^21^.

Dune grass abundance is important in determining dune formation which alters barrier island landscape characteristics. Over the last several decades, the Virginia climate has warmed, resulting in shifting species distributions^19,20^. This warming has likely resulted in an increase in the cover and frequency of *Panicum amarum*, a C_4_ grass. Two decades prior, dunes on Hog were dominated by *Ammophila* and *Spartina*, with *Panicum* only comprising ~ 2% of dune relative cover^37^. By 2022 *Panicum* relative cover increased to > 40% on both Hog and Metompkin which has implications for overall dune structure as *Panicum* exhibits phalanx growth due to shorter rhizomes^39^ and traps 50% less sediment than *Ammophila* or *Spartina*, forming smaller, hummocky dunes. *Panicum* has the potential to alter dune dynamics and growth from those previously documented in Virginia, creating new climate-vegetation scenarios that current models may not predict.

In addition to sediment accretion interactions with grasses, dune building is controlled by large scale geophysical factors that influence sediment availability and abundance^7,9,40^. Even among islands that are geographically close to one another, the abundance and movement of sediment has an influence on island processes^10^ and response to disturbance. In the Virginia barrier island system, both Hog and Metompkin are impacted by similar seasonal weather conditions and disturbance events in the form of hurricanes and nor’easters, and even a moderate storm can have long lasting effects on various island habitats^41^. Storm events can lead to varied impacts with storm surge causing erosion in some places and sediment deposition in others^3,15,16^. Although dune building processes on the two islands are impacted by similar environmental factors, sediment accretion was highest on Hog compared to Metompkin, where several plots transitioned from dune face to open beach or were submerged at high tide as the shoreline has moved landward^8,42^. This is related to a variety of external conditions, including sediment supply and geological processes with Hog having abundant sediment and Metompkin being sediment limited^9,11^. As island-scale changes occur, these factors can determine overall landscape stability.

Species differences emerged when sediment was abundant, as seen on the south end of Hog. In the new dune hummock formation, accretion occurred at a faster rate (35% higher) than in adjacent foredune plots. Accretion may be initially faster in newly developing dunes, but as sediments continue to accrete and as the dune develops, sediment availability is altered and accretion slows^32,43^. As a result of differing disturbance regimes and sediment supply, dune plant cover on Hog was > 40% higher than on Metompkin, with higher stem numbers. On Metompkin, dune cover decreased over time as dunes eroded and transitioned to open beach. These trends in dune vegetative cover can be attributed to frequent disturbance events that cause the ecosystem to reset^44^. Unlike on Hog where succession can continue, plant communities on Metompkin may reset each time a disturbance event occurs, in line with island migration patterns^29,37^.

Dune building directly impacts the interior island swale habitat by providing protection from disturbance and seawater^3,27,29,45^. This connectivity of sediment and seawater (or lack thereof) impacts interior island ecosystem function. In order to relate these ground-obtained metrics to a larger scale, least cost path analysis obtained from digital elevation models was used to compare the ease of water movement from the shoreline into the island interior during 2020 and 2022. Overall, path cost values were highest on Hog compared to Metompkin, aligning with previous conceptual ideas and research^29^ that the dune ridge on Hog is more continuous and robust. Tall, continuous dunes provide increased protection for interior island habitats where successional processes can dominate. The disturbance-moderating effects of these continuous dune ridges also influence interior soil characteristics^29,46^.

Further modeling the influence of water movement across the landscape, storm surge path cost analysis suggests that the tall continuous dune ridge provides substantial protection for the swale habitat. Here, a dune breach is quantified as one single path calculated by ArcGIS Pro through the dune ridge. On Hog, a storm surge of 2.8 m is required to create 3 dune breaches in both 2020 and 2022. Conversely at 2.8 m of storm surge on Metompkin 18 breaches occur in 2020 and 23 occur in 2022. During Hurricane Joaquin in 2015, a maximum storm surge of 1.74 m above mean sea level (1.85 m above NAVD88) caused ecosystem state changes to Virginia barrier islands^41^. In our analysis, breaches were consistently more frequent on Metompkin relative to Hog, demonstrating that a storm will have unequal impacts on barrier islands depending on dune structure. While we are not directly quantifying overwash or storm surge effects on the dune ridge, the ability to estimate at what elevation water will begin to overtop the dune ridge provides an estimate of dune ridge connectivity and swale protection.

The effects of dune height and continuity were observed in the adjacent swales on each island. Higher overwash events on Metompkin were evident based on swale soil chlorides (82% higher than Hog) and bulk density measurements (4% higher). Seasonally higher swale salinity resulted in reduced vegetative cover, ANPP, and soil C and N. As Metompkin experiences higher disturbance, accumulated soil C and N may also be removed as new sand is deposited or leached out of sandy soils^35^. Conversely, on Hog, a salt sensitive, N-fixing shrub (*Morella cerifera*) is expanding range. *Morella* grows in the swale behind protective foredunes, modifies the local grassland microclimate, and inputs C and N into the soils^28,38,47^. The relationship between foredune development and *Morella* expansion may alter nutrient availability, further influencing ecosystem dynamics. Although *Morella* and other woody vegetation are present on Metompkin, expansion has been limited likely due to the enhanced seawater movement into the interior portions of the island, limiting growth^8,48^. Vegetative and soil metrics further reinforce our understanding that there are critical differences in the dune development between the two islands. Although Hog and Metompkin are geographically close and experience similar extreme weather events, dune sediment characteristics are significantly different and lead to impactful changes in the adjacent swale habitat.

## Conclusion

With increases in sea-level rise and storm events, quantifying connectivity of seawater and sediment across the barrier island is essential for predictions of long-term response and resilience. Here we demonstrate the connectivity of adjacent ecosystems (i.e., dune and swale) and how patterns of sediment dynamics alter dune building dynamics, which in turn, influence interior island ecosystem processes. Dunes with higher elevation protect the adjacent interior island from salinity and sediment overwash, allowing for higher vegetative productivity and increased soil C and N. As climate is warming, the increased dominance of *Panicum* (which traps 50% less sediment than other grasses) may reduce the dune building capacity on an island that is already undergoing rapid change (i.e., Metompkin), and impact future response to rising sea-levels. This work demonstrates the importance of accounting for vegetation-sediment interactions and cross-island connectivity into future scenario modeling and predictions that incorporate barrier island habitat.

## Methods

### Study site

**Figure 5:**
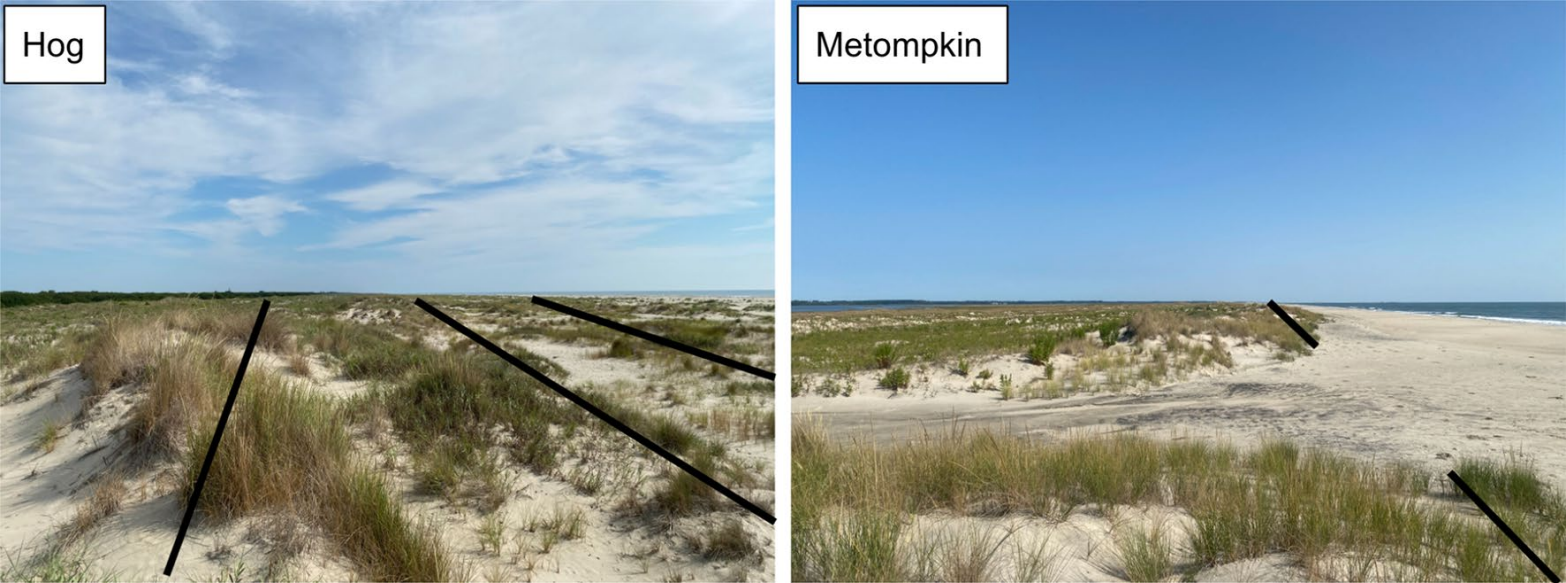
Dune ridges on Hog and Metompkin. On Hog, multiple linear dune ridges are visible, while on Metompkin, dunes are discontinuous and overwash is more prevalent.

This work was focused on Hog Island and Metompkin Island (Fig. 1B), two barrier islands located within the VCR. Both islands are currently undergoing differing responses to disturbance. Hog has experienced minimal island area loss and little conversion of backbarrier marsh to upland over the last ~ 30 years^8^. Recently, sand has been eroding from the north end of the island and depositing on the south end, creating a wide beach allowing for establishment of dune grasses forming new dune hummocks. Hog is characterized by multiple linear dune ridges with swale habitat in between (Fig. 5). The island is on average 1.81 m above the NAVD88 datum^6^. Hog has been characterized as rotationally unstable, where sediment shifts between the northern and southern ends of the island, but the center remains relatively consistent^49^.

Metompkin is frequently disturbed and experiences extensive overwash, causing the island to retreat landward over time^6,8^. Metompkin recently had a linear dune ridge; however, overwash fans have broken through this ridge causing it to be discontinuous^29^ (Fig. 5). Unlike Hog, Metompkin is on average 1.75 m above the NAVD88 datum^6^, and experiences parallel retreat, moving closer to the mainland over time^49^. Further contributing to the disturbance response of Metompkin is downdrift sediment starvation caused by development on islands to the north^11^. This interrupts southward longshore sediment movement, preventing Metompkin from accreting new sediment naturally. Additional contributing factors to the differences between the two islands are ancient geological features that dictate island placement in relation to the mainland. This difference comes in the form of underlying topographic highs around Hog that are absent near Metompkin^9^.

Dominant dune building grasses along the US mid-Atlantic coast include *Ammophila breviligulata, Spartina patens*, and *Panicum amarum*. All are common on the Virginia coast; however, native ranges vary. *Ammophila* is a C_3_ grass limited to more temperate climates with mortality occurring above 35 °C^50^. *Ammophila* is an abundant grass on the Virginia coast with its southern range extending to Cape Fear, NC^51^, although this may be influenced by plantings^20^. *Spartina* and *Panicum* are C_4_ grasses found along the entire US east coast and are abundant on the Virginia coastline^29,52^. Unlike other dune grass species, *Spartina* can thrive in a variety of habitats across the barrier island system, tolerating conditions on the dune ridge, in the swale, and in the back-barrier marsh^44,52^.

*Ammophila* often creates linear dune ridges using lateral rhizomes, resulting in distinct sand accretion and dune stabilization^22,23,33^. Conversely, *Panicum* exhibits phalanx growth (i.e., more spaced-out bunches that do not spread in the same lateral manner) due to shorter rhizome length^22,33,39^. Unlike the other species, dune building patterns of *Spartina* are less documented; however, it has been shown to build dunes, potentially at a slower rate compared to other grasses^31^. Each of these three species also exhibits differing aboveground traits (i.e., height, number of shoots, shoot density, plant density)^39,53^ which may potentially impact sediment accretion.

### Field sampling: vegetation and accretion

To quantify herbaceous species abundance and elevation over time, cross-shore transects were established in August 2020 (n = 3) on the south end of both islands. Transects were spaced 100 m apart and 0.25 m^2^ plots were placed every ~ 5 m from the 2020 high tide line spanning the beach, dune, and into the swale, stopping prior to a shrub thicket when present (n = 30). At each plot, location and elevation were recorded with Trimble R10-2 and TSC7 high resolution GPS receivers (Trimble Inc., Westminster, CO). In August 2020, 2021, and 2022, plots were quantified for percent cover by species. Seasons end biomass was sampled in a 10 × 100 cm plot adjacent to each composition plot to quantify aboveground annual net primary productivity (ANPP). Due to logistical constraints, biomass was not collected on Hog in August 2022.

In November 2021, additional sampling plots were established on the south ends of both islands within the area spanning the existing transects, located on the foredune and in the swale. To quantify species interactions with sediment and soil characteristics, 0.25 m^2^ plots were established based on species presence in naturally occurring monocultures (i.e., *Ammophila, Spartina, Panicum*, n = 5) on the foredune face. On Hog, additional plots were placed in the new dune hummock formation for *Ammophila*, *Spartina*, *Panicum* (n = 5). Within the swale, plots were located behind dunes in mixed species grassland (n = 15). Snow poles (123 cm in height and 0.8 cm in diameter) were installed in the center of each plot to monitor sediment accretion. Poles were driven into the ground, leaving ~ 70 cm above the soil surface. Baseline measurements of exposed pole heights were obtained using a meter stick to the nearest mm to indicate starting sand level at each sample location. Seasonal measurements of sediment accretion, species cover, stem density, and soil characteristics (described below) were conducted in 2021 (November) and 2022 (March, August, and November). Species percent cover and stem count were quantified within the 0.25 m^2^ plot.

### Field sampling: soil characteristics

Soil cores were obtained directly outside of the 0.25 m^2^ plot using a 30 cm metal tube and a mallet (n = 5 at each dune location per species, per island, per season; n = 5 in swale per island, per season). Height of the soil core was measured in the field, and cores were stored in soil collection bags to be transported back to the lab for processing. Bulk density was quantified to determine soil compaction by measuring the volume of soils in the field, and dry weight of the soil samples after drying at 100 °C for 48 h. Soil organic matter content was measured using the loss on ignition method by placing 1 g dry soil in a muffle furnace at 550 °C for 6 h. Additional samples were sent to the Cornell Isotope Lab for additional analysis of total C and N content. Soil chlorides were quantified to assess salinity content using an Orion Research digital ion analyzer (model: 501, Orion Corporation, Espoo, Finland) to measure the conductivity (mV) of each sample and compare to known saline concentrations.

### Aerial imagery and geographic information systems

To quantify ease of sediment/water movement across the landscape, we produced aerial orthomosaic and digital elevation models (DEM) in 2020 and 2022 using aerial imagery collected from 100 m altitude using a DJI Phantom 4 Pro RTK unoccupied aerial system (UAS), (SZ DJI Technology Co., Ltd., Shenzhen, China). The resulting imagery had a resolution of approximately 3 cm pixel^−1^ with a relative horizontal precision of 0.4 cm and a vertical uncertainty of 1.2 cm for Hog, and a relative horizontal uncertainty of 0.2 cm and a vertical uncertainty of 0.8 cm for Metompkin. Imagery was processed from images with 80% forward and 70% side overlap along the programmed UAS flight paths.

We processed raw UAS imagery using Agisoft Metashape Pro version 1.7 (Agisoft, St. Petersburg, Russia) into point clouds from which the final orthomosaics and DEMs were derived using structure-for-motion and tiling processes. These (Fig. S4) were georeferenced during processing in Metashape Pro with ground control points (2-m steel rebar lengths driven into the ground) captured in the UAS imagery using 1-m^2^ black and white targets placed at each of the 5 points/island. Control points were surveyed in 2019 with a Trimble R10 RTK system (4.9 cm horizontal uncertainty, 9.8 cm vertical uncertainty on Hog; 1.5 cm horizontal uncertainty, 4.2 cm vertical uncertainty on Metompkin).

A range of different methods exist to predict water dynamics and sediment movement on coastal beaches. Numerous process-based models exist to simulate factors like wave setup, swash processes, beach and dune evolution, dune breaching, and overwash processes^54–56^. XBeach^57^ is among the most widely used tools for storm forecasting of overwash processes, although models such XBeach have been shown to be highly sensitive to pre-storm bathymetric inputs^58^; data which are not available for this field study. Tools such as empirical equations have less computational cost and fewer site-specific data requirements and are also widely used to calculate either the vertical extent of wave runup onto the beach and related erosional processes to dune systems^59,60^. However, these empirical approaches are only one-dimensional in the cross-shore direction and do not account for two-dimensional (2D) flow effects. As an intermediate method that can characterize possible 2D water and sediment patterns across the beach/dune system into the swale, we implemented a GIS-based method using least cost path analysis.

To determine topographic factors impacting dune-swale connectivity, DEM imagery analysis was performed using ArcGIS Pro version 2.8.3 (Esri, Redlands, CA). Least cost path analysis was quantified on each island to determine the potential for water and sediment movement into the swale. Least cost path analysis is a useful tool in determining where topographical barriers exist within a landscape. This metric (path cost value) is most commonly used when assessing watersheds in mainland areas, however, here we use it as a measure of how continuous dune protection is on the barrier island. While the interpretation of least cost path analysis when utilized in this way will differ from traditional usages, it has been demonstrated to provide valuable insight into topographical resistance in barrier island and dune systems^27,61–65^. The path cost value measures the amount of resistance caused by elevation changes moving from designated start/end points. Least cost path was evaluated starting on the beach (near the high-water mark) to the first swale behind the primary linear dune ridge (n = 500; Fig. S5). Higher path cost values indicated more resistance to seawater flow across the dune system.

Additional analysis was performed to determine what level of overwash is required to breach the primary dunes. Dunes were extracted by elevation, starting with 2.0 m above the NAVD88 datum, and increasing by 0.20 m up to 3.6 m as the increase in number of paths was reduced. The starting elevation was set at 2.0 m to reflect the mean dune elevation recorded on Hog Island (2.33 ± 0.11 m). Intervals of 0.20 m were chosen to reduce computation time when running the analysis in ArcGIS Pro. The number of potential paths across the landscape provides an idea as to how continuous the dune ridge remains at different water levels. Path costs were also calculated including dunes as objects, allowing for the determination of path costs values for both islands at different storm surge levels (Fig. S6).

### Statistics

For statistical analysis, dune and swale habitats were considered separately. In order to meet assumptions of normality, percent cover, stem count, organic matter, ANPP, and chlorides were log + 1 transformed. In the dune habitat, percent cover, stem density, accretion, and soil metrics were analyzed via 3-way ANOVA with habitat locations, species, and season as treatment variables. In the swale habitat, percent cover, ANPP, and soil metrics were analyzed via 2-way ANOVA with island and season as treatment variables. Tukey’s Honest Significant Difference tests were performed on significant interactions or main effects of ANOVA tests. Correlations were utilized to determine relationships between sediment accretion and biotic variables mentioned. Path cost values and Trimble elevations were analyzed using a 2-way ANOVA with island and year as treatment variables. Soil C and N percentages were not normally distributed and were analyzed using Wilcoxon Rank Sums tests. Analyses were completed using JMP Pro statistical software version 16.1.0 (SAS, Cary, NC).

### Supplementary Information

Supplementary Information.

## Data Availability

Data are available at https://www.vcrlter.virginia.edu/cgi-bin/showDataset.cgi?docid=knb-lter-vcr.392.2 and 10.6073/pasta/98d5c6ba4e82cca8ffc12e3c0922a668.
