# Supplementary material for: Drivers of dune formation control ecosystem function and response to disturbance in a barrier island system
Source: Sci Rep. 2024 May 18;14:11405. doi: 10.1038/s41598-024-61741-9 (PMC11102538; doi:10.1038/s41598-024-61741-9)
Supplement: Supplementary file 1 — Supplementary Information. [file 41598_2024_61741_MOESM1_ESM.docx]

**Supplementary Tables and Figures**

Table S1. Three-way ANOVA results for sediment accretion rate (cm month^-1^) by season, dominant dune grass species, and island. Significant factors are shown in bold.

| Source |  | F | *p* |
| --- | --- | --- | --- |
| Season |  | 0.0367 | 0.9639 |
| Dominant Species |  | 0.8391 | 0.4363 |
| **Island** |  | **5.8823** | **0.0178** |
| Season*Dominant Species |  | 0.0761 | 0.9893 |
| Season*Island |  | 1.3445 | 0.2671 |
| Dominant Species*Island |  | 0.3261 | 0.7228 |
| Season*Dominant Species*Island |  | 0.0692 | 0.9911 |

Table S2. Three-way ANOVA results for sediment accretion rate (cm month^-1^) on Hog by season, habitat, and island. Significant factors are shown in bold.

| Source |  | F | *p* |
| --- | --- | --- | --- |
| **Season** |  | **23.4416** | **<0.0001** |
| **Habitat** |  | **12.6978** | **0.0007** |
| **Dominant Species** |  | **12.0853** | **<0.0001** |
| Season*Habitat |  | 2.7746 | 0.0691 |
| Season*Dominant Species |  | 1.862 | 0.1664 |
| Habitat*Dominant Species |  | 0.0077 | 0.9924 |
| Season*Habitat*Dominant Species |  | 0.2422 | 0.7859 |

Table S3. Three-way ANOVA results for percent cover by season, dominant dune grass species, and island. Significant factors are shown in bold.

| Source |  | F | *p* |
| --- | --- | --- | --- |
| **Season** |  | **9.0867** | **<0.0001** |
| Dominant Species |  | 0.3416 | 0.7115 |
| **Island** |  | **43.5384** | **<0.0001** |
| Season*Dominant Species |  | 0.3726 | 0.8948 |
| **Season*Island** |  | **11.1973** | **<0.0001** |
| Dominant Species*Island |  | 1.2564 | 0.2893 |
| Season*Dominant Species*Island |  | 0.1493 | 0.9888 |

Table S4. Three-way ANOVA results for stem count by season, dominant dune grass species, and island. Significant factors are shown in bold.

| Source |  | F | *p* |
| --- | --- | --- | --- |
| Season |  | 1.8481 | 0.1667 |
| **Dominant Species** |  | **5.9413** | **0.0045** |
| **Island** |  | **12.5352** | **0.0008** |
| Season*Dominant Species |  | 1.6662 | 0.1702 |
| Season*Island |  | 2.2204 | 0.1177 |
| Dominant Species*Island |  | 2.0149 | 0.1426 |
| Season*Dominant Species*Island |  | 0.1869 | 0.9443 |

Table S5. Two-way ANOVA results for ANPP (gm^-2^ yr^-1^) in the dune habitat by year and island. Significant factors are shown in bold.

| Source |  | F | *p* |
| --- | --- | --- | --- |
| Year |  | 0.2402 | 0.6276 |
| Island |  | 0.3337 | 0.5678 |
| Year*Island |  | 0.0071 | 0.9333 |

Table S6. Three-way ANOVA results for organic matter in the dune habitat by season, dominant dune grass species, and island. Significant factors are shown in bold.

| Source |  | F | p |
| --- | --- | --- | --- |
| Season |  | 1.5936 | 0.2105 |
| Dominant Species |  | 0.8905 | 0.4151 |
| Island |  | 0.0025 | 0.9603 |
| Season*Dominant Species |  | 1.4530 | 0.2258 |
| **Season*Island** |  | **4.4362** | **0.0153** |
| Dominant Species*Island |  | 3.2269 | 0.0457 |
| Season*Dominant Species*Island |  | 0.8785 | 0.4813 |

Table S7. Three-way ANOVA results for soil chlorides (μg g^-1^) in the dune habitat by season, dominant dune grass species, and island. Significant factors are shown in bold.

| Source |  | F | *p* |
| --- | --- | --- | --- |
| **Season** |  | **15.6630** | **<0.0001** |
| Dominant Species |  | 0.5018 | 0.6076 |
| **Island** |  | **61.0270** | **<0.0001** |
| **Season*Dominant Species** |  | **3.1593** | **0.0191** |
| **Season*Island** |  | **8.0657** | **0.0007** |
| Dominant Species*Island |  | 0.4347 | 0.6492 |
| Season*Dominant Species*Island |  | 1.1075 | 0.3600 |

Table S8. Three-way ANOVA results for bulk density (g cm^-3^) in the dune habitat by season, dominant dune grass species, and island. Significant factors are shown in bold.

| Source |  | F | p |
| --- | --- | --- | --- |
| **Season** |  | **140.3707** | **<0.0001** |
| Dominant Species |  | 0.0691 | 0.9333 |
| **Island** |  | **19.0211** | **<0.0001** |
| Season*Dominant Species |  | 1.4527 | 0.2259 |
| Season*Island |  | 1.0055 | 0.3711 |
| Dominant Species*Island |  | 0.8120 | 0.4481 |
| Season*Dominant Species*Island |  | 1.9579 | 0.1105 |

Table S9. Three-way ANOVA results for elevation (m) by habitat, island, and year. Significant factors are shown in bold.

| Source |  | F | *p* |
| --- | --- | --- | --- |
| **Habitat** |  | **4.6658** | **0.0316** |
| **Island** |  | **25.9781** | **<0.0001** |
| Year |  | 0.9156 | 0.4015 |
| **Habitat*Island** |  | **4.3234** | **0.0385** |
| Habitat*Year |  | 2.2420 | 0.1081 |
| **Island*Year** |  | **6.9928** | **0.0011** |
| Habitat*Island*Year |  | 1.5599 | 0.212 |

Table S10. Two-way ANOVA results for path cost value by island and year. Significant factors are shown in bold.

| Source |  | F | *p* |
| --- | --- | --- | --- |
| **Island** |  | **133.0454** | **<0.0001** |
| **Year** |  | **159.7671** | **<0.0001** |
| **Month*Island** |  | **113.2582** | **<0.0001** |

Table S11. Three-way ANOVA results for storm surge path cost analysis. Significant factors are shown in bold.

| Source |  | F | *p* |
| --- | --- | --- | --- |
| **Island** |  | **855.0903** | **<0.0001** |
| Year |  | 11.0069 | 0.0009 |
| **Storm surge elevation** |  | **12.5707** | **<0.0001** |
| **Island*Year** |  | **24.0075** | **<0.0001** |
| **Island*Storm surge elevation** |  | **8.1386** | **<0.0001** |
| Year*Storm surge elevation |  | 0.1583 | 0.9244 |
| Island*Year*Storm surge elevation |  | 0.1856 | 0.9062 |

Table S12. Two-way ANOVA results for plant cover in the swale habitat by season and island. Significant factors are shown in bold.

| Source |  | F | *p* |
| --- | --- | --- | --- |
| **Season** |  | **2.9458** | **0.0413** |
| **Island** |  | **45.2111** | **<0.0001** |
| Season*Island |  | 0.1122 | 0.9526 |

Table S13. Two-way ANOVA results for ANPP (gm^-2^ yr^-1^) in the swale habitat by year and island. Significant factors are shown in bold.

| Source |  | F | *p* |
| --- | --- | --- | --- |
| Year |  | 0.0319 | 0.8585 |
| **Island** |  | **10.3035** | **0.0016** |
| Year*Island |  | 0.9894 | 0.3215 |

Table S14. Two-way ANOVA results for organic matter in the swale habitat by season and island. Significant factors are shown in bold.

| Source |  | F | *p* |
| --- | --- | --- | --- |
| Season |  | 0.3944 | 0.6751 |
| **Island** |  | **6.3844** | **0.0185** |
| Season*Island |  | 0.9090 | 0.4164 |

Table S15. Two-way ANOVA results for chlorides (μg g^-1^) in the swale habitat by season and island. Significant factors are shown in bold.

| Source |  | F | *p* |
| --- | --- | --- | --- |
| **Season** |  | **7.1718** | **0.0022** |
| **Island** |  | **9.4438** | **0.0039** |
| **Season*Island** |  | **4.4085** | **0.0188** |

Table S16. Two-way ANOVA results for bulk density (g cm^-3^) in the swale habitat by season and island. Significant factors are shown in bold.

| Source |  | F | *p* |
| --- | --- | --- | --- |
| **Season** |  | **85.4091** | **<0.0001** |
| **Island** |  | **13.3786** | **0.0008** |
| Season*Island |  | 2.6898 | 0.0805 |


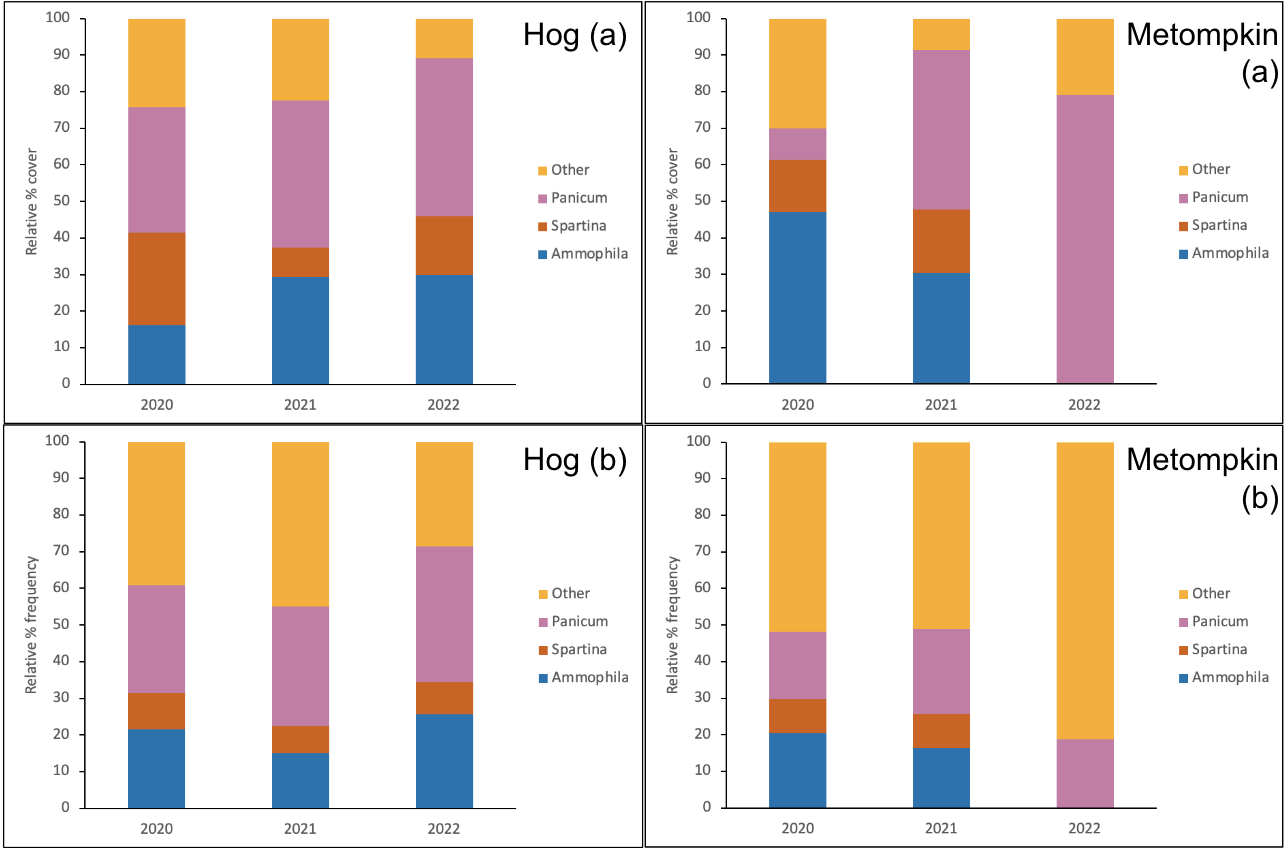


Figure S1. Relative % cover (a) and relative % frequency (b) for dune grass species along cross-island transects on Hog and Metompkin. Species included in ‘other’ category include: *Conyza canadensis*, *Solidago sempervirens*.


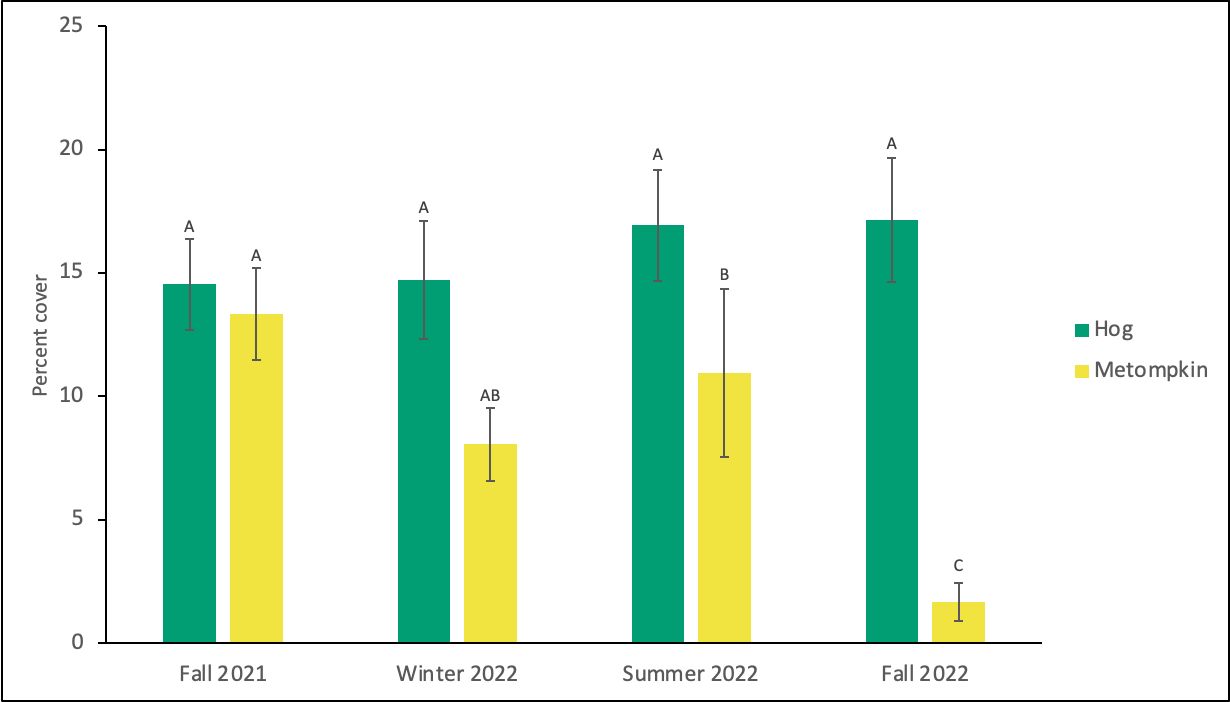


Figure S2. Average percent cover of dominant dune grasses by season on Hog and Metompkin. Letters indicate statistical 2-way interaction differences, error bars show standard error. Cover is significantly lower on Metompkin, while remaining relatively high on Hog.


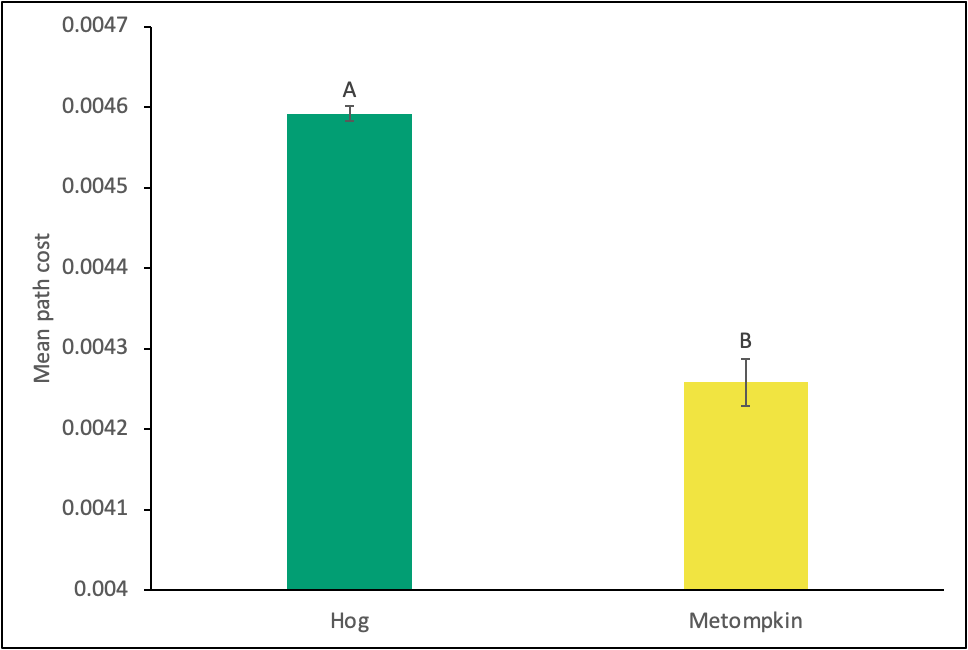


Figure S3. Average path cost for Hog and Metompkin. Error bars represent standard error, letters indicate significant difference.


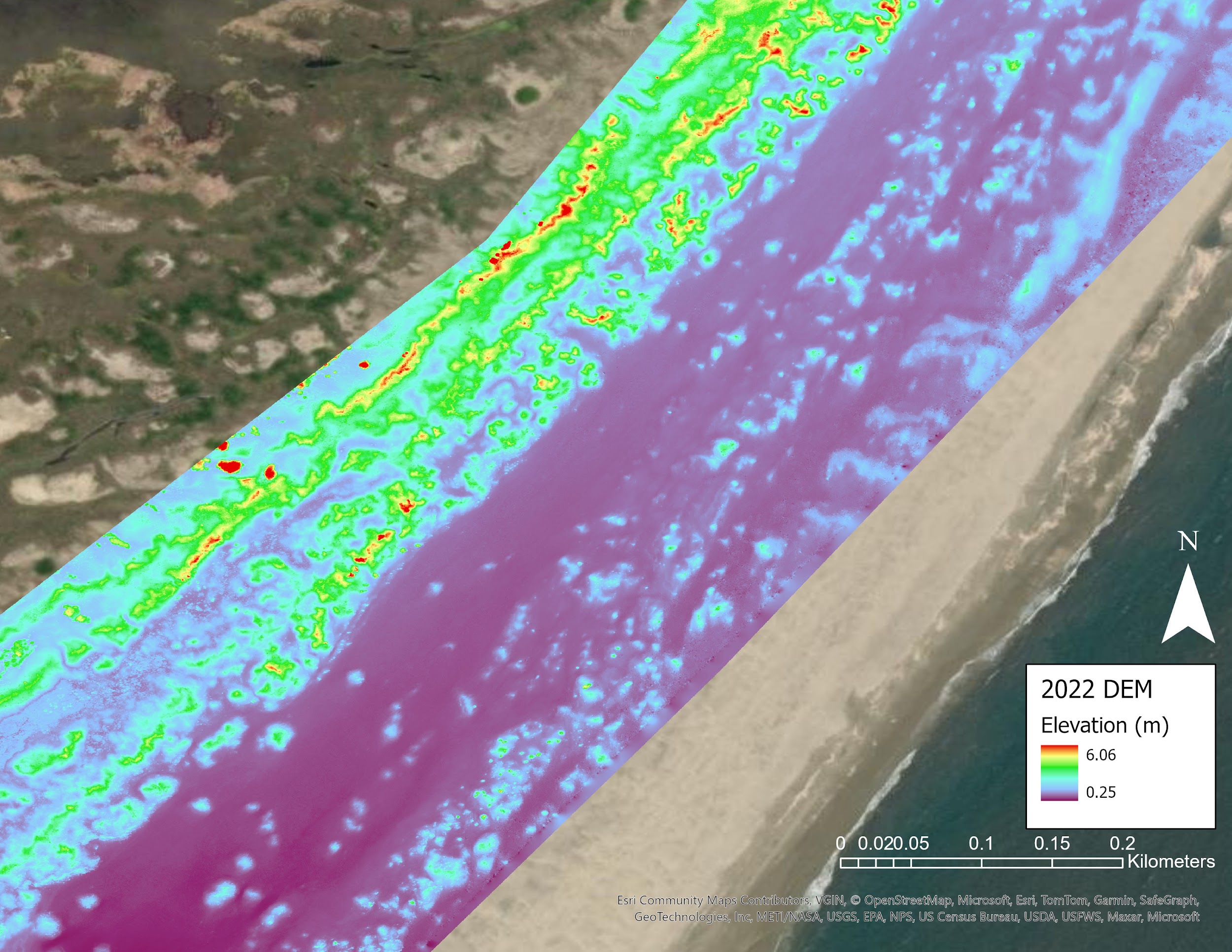


Figure S4. Digital elevation model overlaid on Hog Island. Elevation (m) relative to NAVD88 datum.


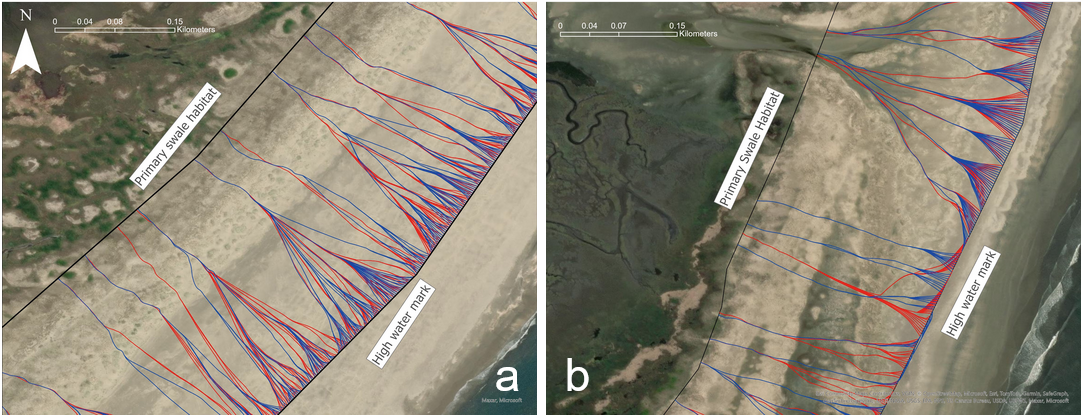
Figure S5. Visual representation of least-cost paths across Hog (A) and Metompkin (B) Islands. 2020 paths are shown in red, 2022 paths shown in blue.


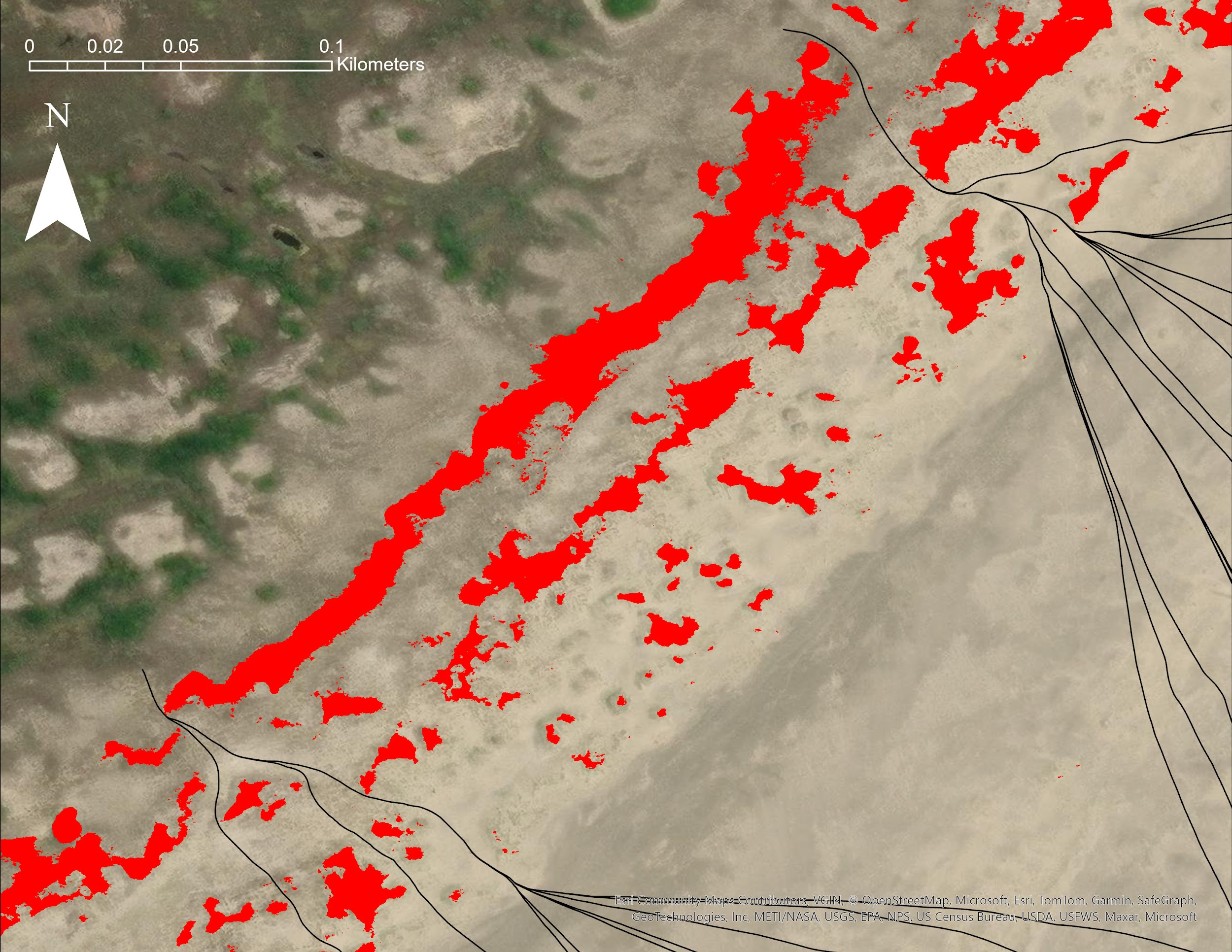


Figure S6. Path cost analysis represented for storm surge assessment at 3.0 m on Hog 2022. Dunes over 3.0 m are shown in red, optimal paths across the landscape are shown in black lines.
